# Infectious complications in children with acute myeloid leukemia: decreased mortality in multicenter trial AML-BFM 2004

**DOI:** 10.1038/bcj.2015.110

**Published:** 2016-01-15

**Authors:** K Bochennek, A Hassler, C Perner, J Gilfert, S Schöning, T Klingebiel, D Reinhardt, U Creutzig, T Lehrnbecher

**Affiliations:** 1 Division of Pediatric Hematology and Oncology, Hospital for Children and Adolescents, Johann Wolfgang Goethe-University, Frankfurt, Germany; 2 Paediatric Hematology and Oncology, Medical Center, University of Essen, Essen, Germany; 3 Department of Pediatric Hematology and Oncology, Children's Hospital, Hannover Medical School, Hannover, Germany

## Abstract

Infections are an important cause for morbidity and mortality in pediatric acute myeloid leukemia (AML). We therefore characterized infectious complications in children treated according to the trial AML-BFM 2004. Patients with Down syndrome were excluded from the analysis. Data were gathered from the medical records in the hospital where the patients were treated. A total of 405 patients (203 girls; median age 8.4 years) experienced 1326 infections. Fever without identifiable source occurred in 56.1% of the patients and clinically and microbiologically documented infections in 17.5% and 32.4% of the patients, respectively. In all, 240 Gram-positive (112 viridans group streptococci) and 90 Gram-negative isolates were recovered from the bloodstream. Invasive fungal infection was diagnosed in 3% of the patients. Three children each died of Gram-negative bacteremia and invasive aspergillosis, respectively. As compared with the results of AML-BFM 93 with lower dose intensity, infection-related morbidity was slightly higher in AML-BFM 2004 (3.3. versus 2.8 infections per patient), whereas infection-related mortality significantly decreased (1.5% versus 5.4% *P*=0.003). Specific anti-infective recommendations included in the treatment protocol, regular training courses for pediatric hematologists and increasing experience may be the reason for reduced infection-related mortality in children with AML. Further studies are needed to decrease infection-related morbidity.

## Introduction

Over the past decade, several studies reported that children with acute myeloid leukemia (AML) are at a particularly high risk for infectious complications related to the highly intensive chemotherapy, resulting in prolonged severe neutropenia and the profound disruption of other arms of the immune system.^1, 2, 3^ Infections not only contribute to mortality, but also prolong hospitalization, delay the administration of chemotherapy, decrease quality of life and require the administration of costly and often toxic antimicrobial compounds. Because of the high infection-related morbidity and mortality in children with AML, a variety of supportive care measures have been proposed, but are still controversially discussed.^4, 5, 6, 7, 8, 9, 10^

The main objective of study AML-BFM 2004 (ClinicalTrials.gov NCT00111345) was the improvement of prognosis by intensification of chemotherapy, but at the same time avoiding unacceptably high toxicity.^11^ In this regard, induction therapy randomly evaluated liposomal daunorubicin (L-DNR) in a dose higher than the assumed equivalent dose of idarubicin. In addition, intensification of consolidation for high-risk (HR) patients with 2-chloro-2-deoxyadenosine (2-CDA) was investigated. To improve supportive care measures, specific recommendations for infection-related diagnostics and for antimicrobial prophylaxis and treatment were included in the protocol, but were not mandatory. In addition, regular training courses regarding infectious complications have been implemented for pediatric hematologists in Germany since 1999.

The objective of the present analysis was to describe incidence and characteristics of infectious complications and infectious deaths in children treated according to the trial AML-BFM 2004. Results were compared with the data of study AML-BFM 93 that we have reported previously.^1^

## Patients and methods

Of a total of 466 patients enrolled in the prospective multi-institutional clinical trial AML-BFM 2004, data on infectious complications during intensive treatment were gathered by two of the authors (CP and JG) in the hospital where the patients had been treated. Infectious complications were defined as clinical signs and symptoms associated with the institution of antibiotic therapy, the isolation of a pathogen or an identifiable site of infection by physical examination or imaging study, respectively.^1^ Infectious episodes were categorized as fever of unknown origin, as microbiologically documented or as clinically documented infection. One cycle of chemotherapy was defined as the time from the start of chemotherapy until the day before the start of the next cycle of chemotherapy. Fever was considered as temperature >38.5 °C once or 38–38.5 °C twice within a 4-h interval, and neutropenia as an absolute neutrophil count 500/*μ*l. Bacteremia was defined as fever with a positive blood culture for bacteria isolated from peripheral blood or from the central venous indwelling catheter. If the bloodstream isolate was a potential skin contaminant (for example, coagulase-negative staphylococcus), the presence of an intravascular catheter was required for the diagnosis of a bloodstream infection.^12^ Infection of the gastrointestinal tract required the association of clinical symptoms with a pathogen (such as *Clostridium difficile*), whereas diarrhea alone or the recovery of a pathogen in the stool without clinical symptoms did not fulfill the criteria of gastrointestinal tract infection. The diagnosis of pneumonia required a pathological chest X-ray and/or computed tomography scan accompanied by clinical symptoms of lower respiratory infection. Invasive fungal disease was defined according to the revised definitions of the EORTC/MSC (European Organization for Research and Treatment of Cancer/Invasive Fungal Infections Cooperative Group and the National Institute of Allergy and Infectious Diseases Mycoses Study Group) consensus group.^13^

Supportive care was administered according to the discretion of the treating physician and institutional standards, although detailed recommendations such as for antimicrobial prophylaxis or for empirical therapy were included in the study protocol. Specifically, anti-mold active prophylaxis was recommended (for example, using voriconazole), whereas no general recommendations regarding antibacterial prophylaxis (for example, using penicillin) were given because of the lack of firm data. The importance of early empiric therapy was underlined, and first-line use of a glycopeptide in severely ill patients was recommended. Regular training courses regarding infectious complications were instituted for centers participating in the BFM studies since 1999, where pros and cons of different strategies were discussed.

### Treatment

In the multicenter clinical trial AML-BFM 2004, which was performed between March 2004 and April 2010, patients were stratified into either the standard-risk (SR) or the high-risk (HR) group.^11^ For intensive chemotherapy, both risk groups received induction therapy that consisted of cytarabine (100 mg/m^2^/day continuous infusion on days 1 and 2, followed by 30 min infusion twice daily on days 3–8), etoposide (150 mg/m^2^/day on days 6–8) in combination with L-DNR (80 mg/m^2^/day for 3 days; ADxE) or idarubicin (12 mg/m^2^/day for 3 days; AIE) as randomized. Only HR patients received a second induction with high-dose cytarabine 3 g/m^2^/day for 3 days and mitoxantrone 10 mg/m^2^/day for 2 days (HAM). In HR patients, regular consolidation (AI) consisting of 500 mg/m^2^/day cytarabine for 4 days of continuous infusion plus 7 mg/m^2^/day idarubicin for days 3 and 5 was randomly compared with intensification adding 2-CDA (6 mg/m^2^/day for 2 days; AI/2-CDA), whereas SR patients received AI. Second induction with AI in SR patients and consolidation in HR patients, respectively, was followed by a short chemotherapy cycle with 0.5 g/m^2^/day cytarabine (4 days) and idarubicine 7 mg/m^2^/day for 2 days (haM). All patients received intensification (HAE) with high-dose cytarabine (3 g/m^2^ every 12 h for 3 days) and etoposide (125 mg/m^2^/day for 4 days).

### Statistical analysis

Data were analyzed using GraphPad Prism (GraphPad Prism Software version 5.04 for Windows, GraphPad Software, La Jolla, CA, USA, www.graphpad.com). Comparisons of different groups were performed using the χ^2^ test or Fisher's exact test, when applicable. A *P*-value (two tailed) of <0.05 was considered to be statistically significant.

## Results

### Patient characteristics

Data on infectious complications were collected from 466 patients representing 76.3% of all 611 patients enrolled in the multicenter prospective clinical trial AML-BFM 2004. As patients with AML and Down syndrome receive a less-dose-intensive chemotherapeutic regimen, the 61 patients diagnosed with Down syndrome were excluded from the analysis and will be reported elsewhere. The remaining 405 patients (202 boys and 203 girls) had a median age of 8 years 4 months (range, 2 days–17 years 11 months). In all, 118 patients were treated according to SR (induction therapy: 56 patients randomized for ADxE, 62 patients for AIE), 287 according to HR (induction therapy: 142 patients randomized for ADxE, 145 patients for AIE; consolidation: 123 patients randomized for AI, 138 patients for AI-2CDA). The patients were treated in 37 centers (4–28 patients per center). The FAB (French–American–British) classification of the patients was as follows: 13 patients with M0, 54 with M1, 89 with M2, 26 with M3, 88 with M4, 89 with M5, 10 with M6 and 31 with M7; 5 patients could not be classified.

Nearly all patients (96.8%) had an indwelling central venous catheter, and 98.2% were receiving trimethroprim–sulfamethoxazole prophylaxis for *Pneumocystis jiroveci*. Antibacterial prophylaxis, which was not further specified, and anti-mold active antifungal compounds were given in 26.3% and 71.4% of the chemotherapy cycles, respectively.

### Infectious complications

During intensive treatment, the 405 patients experienced a total of 1326 infectious episodes (3.3 infectious episodes per patient). Patients treated according to HR had slightly more infectious complications per patient than patients treated according to SR (3.3 versus 3.1; not significant). Whereas 20 patients did not experience any infectious complication, one infection occurred in 44 patients, two in 61, three in 99, four in 94, five in 48, six in 32, seven in 2, eight in 4 and nine in 1 of the patients, respectively. Out of the 1326 infectious episodes, fever of unknown origin was diagnosed in 744 episodes (56.1%), and clinically and microbiologically documented infections in 152 and 430 episodes, respectively (17.5% and 32.4%, respectively) (Tables 1a and b). The distribution of fever of unknown origin and clinically and microbiologically documented infections did not differ between patients treated according to SR and HR (58.4%, 11.7% and 29.9% versus 55.2%, 11.4% and 33.4%, respectively). The randomized chemotherapy cycles (ADxE versus AIE, and AI-2CDA versus AI, respectively) did not significantly differ regarding infectious complications. In both risk groups, neutropenia was present in ∼80% of the infection episodes (79% in SR and 81.8% in HR, respectively).

Among the 430 microbiologically documented infections, 308 bacteremia occurred (71.6%). Overall, 240 Gram-positive (72.7%) and 90 Gram-negative (27.3%) isolates were recovered from the bloodstream, most of them in neutropenic patients (166 (69.2%) Gram-positive and 72 (80%) Gram-negative pathogens; Tables 2a and b). In 19 episodes (5.8%), more than one pathogen was isolated. For both SR and HR patients, Gram-negative pathogens were significantly more often isolated in consolidation and intensification as compared with induction (SR patients: 19/211 chemotherapy cycles haM and HAE versus 3/228 cycles of induction (*P*<0.001); HR patients: 55/723 chemotherapy cycles haM and HAE versus 13/573 chemotherapy cycles of induction (*P*<0.01)). A significant preponderance of Gram-positive pathogens was also seen in HR patients for consolidation and intensification (*P*<0.001), whereas in SR patients, the incidence of Gram-positive pathogens was evenly distributed across the cycles of chemotherapy. Viridans group streptococci (VGS) were recovered in 37.9% of and 32.5% of bloodstream infections in SR and HR patients, respectively. However, no overrepresentation of VGS was seen in chemotherapy cycles that included high-dose cytarabine (HAE in SR and HAM and HAE in HR, respectively).

Invasive fungal infections were diagnosed in a total of 12 patients (3%). Specifically, *Candida* infection occurred in two patients of the SR group (one patient with candidemia and one patient with chronic disseminated candidiasis). Probable invasive pulmonary aspergillosis was diagnosed in eight patients (three patients of the SR group and five patients of the HR group), and pulmonary aspergillosis was proven in another two patients of the HR group. Of the 12 fungal infections, 9 occurred during induction therapy.

Next to bloodstream infections, pneumonia was the most common site of infection (Tables 3a and b). The incidence of pneumonia was significantly higher in induction therapy compared with the other chemotherapy cycles (SR: ADxE/AIE versus AI, haM and HAE, respectively; *P*=0.009; HR: ADxE/AIE and HAM versus consolidation, haM and HAE, respectively; *P*<0.001). In contrast, gastrointestinal, soft tissue and urinary tract infections were evenly distributed across the chemotherapy cycles.

A total of 55 patients (13.6%) needed intensive care treatment, mostly during induction therapy (31/55 (56.4%)). The main reasons for intensive treatment were pneumonia and/or sepsis due to VGS (11 patients (20%)), sepsis due to Gram-negative bacteria (6 patients (10.9%)) and pneumonia without causative pathogen identified (17 patients (30.9%)).

### Infection-related mortality

Overall, 6 of the 405 patients (1.5% median age (range) 11 years (1 month to 12 years)) died due to infectious complications. Three of the patients were in remission, and death occurred in three patients before day 42 (‘early death'). The median time (range) of death was 58 days (24–160) after diagnosis of AML. In three patients each, death was due to bacterial infection (*Salmonella* species, *P*seudomonas *aeruginosa* and *Stenotrophomonas maltophilia* in one patient each) and due to invasive aspergillosis, respectively. Notably, no patient died because of VGS.

## Discussion

In the multi-institutional clinical trial AML-BFM 2004, 3.3 infectious complications per patient occurred during intensive treatment. Almost three-quarters of the bloodstream infections were due to Gram-positive bacteria, and more than 30% were due to VGS. Invasive fungal infection was diagnosed in only 3% of the patients. Overall, 1.5% of the patients died because of infections, all of them due to Gram-negative pathogens or invasive aspergillosis. No patient died because of VGS.

In the present study, the incidence of infectious complications was slightly higher compared with the study AML-BFM 93 that was less dose intense (3.3. versus 2.8 infectious episodes per patient).^1^ In line with other reports, the majority of children with AML experienced at least one infectious episode, and most of them occurred during neutropenia.^1, 2, 14^ Among bloodstream infections, Gram-positive episodes were predominant, as it has also been demonstrated by others.^1, 2, 14^ The incidence of VGS isolated in one-third of all bloodstream infections was unexpectedly and considerably higher than observed by Sung *et al.*^1, 2, 15^ in CCG 2961 and observed in our previous analysis of AML-BFM 93 in which VGS accounted for ∼20% of pathogens isolated in the bloodstream. The reason for this important finding remains unclear, but it has recently been reported that quinolone prophylaxis significantly increased the incidence to VGS bacteremia in children with AML.^16^ In our analysis, antibacterial prophylaxis was given in one-quarter of all chemotherapy cycles, but unfortunately we did not specify the exact antibacterial compound in our data base, preventing any further conclusion. The optimal strategies to prevent VGS bacteremia have yet to be defined and are a matter of controversy.^5, 6, 7, 8, 9, 10^ Importantly, despite the high incidence of VGS bacteremia in the present study, only 11 out of 112 patients (9.8%) needed intensive care treatment due to VGS sepsis, and no patient died because of VGS, suggesting the high vigilance of treating physicians with prompt institution of adequate treatment. In this regard, the Canadian Infections in AML Research Group reported that 11.2% children with AML and VGS bacteremia had to be transferred to the intensive care unit.^15^

The relatively low number of proven and probable invasive fungal infections in study AML-BFM 2004 may be explained, at least in part, by the fact that antifungal prophylaxis was administered in >70% of the chemotherapy cycles, mostly with anti-mold active compounds such as amphotericin B, voriconazole or posaconazole. A higher incidence of invasive fungal infection in pediatric AML was described in the clinical trial CCG2961 by the Children's Cancer Group (CCG), but a survey demonstrated a more restricted use of antifungal prophylaxis by these centers as compared with BFM centers.^2, 17^ However, it has to be noted that diagnosing invasive fungal infection in children is particularly challenging, and experts of the EORTC/MSG consensus group are currently revising the definitions of invasive fungal infections for this age group.^18^

In the present trial, infection-related mortality was significantly lower than in our previous report on study AML-BFM 93 (1.5% versus 5.4% in patients without Down syndrome, *P*=0.003).^1^ Compared with the data of AML-BFM 2004, higher rates of infection-related mortality were also reported in 525 children treated according to protocols of the Nordic Society of Paediatric Haematology and Oncology (NOPHO) between 1984 and 2003 and in 492 children enrolled in CGC 2961 of the Children's Cancer Study Group (infection-related mortality 9% and 11%, respectively).^2, 19^ As AML-BFM 93 was less dose intense than AML-BFM 2004, other factors than chemotherapy intensity are likely to contribute to the lower infection-related mortality. In this regard, because of the high infection-related mortality in trial AML-BFM 93, we have included extensive recommendations for diagnostic procedures regarding infectious complications, antifungal prophylaxis and the prompt institution of empiric therapy that should include a glycopeptide as first line in severely ill patients in the protocol AML-BFM 2004, although these recommendations were not mandatory. In addition, since 1999, regular training courses were implemented in the education of pediatric hematologists in Germany and may have helped to decrease infection-related mortality in children with AML. Finally, the treating centers became more familiar with managing infectious complications in children with AML, and this was also reported as contributing factor for the substantial decline of infection-related mortality in the study MRC-10.^20^

In conclusion, this is the first comparative analysis on infection-related morbidity and mortality in two different studies on pediatric AML performed by the same cooperative group over two distinct periods of time. Our results demonstrate that the infection-related mortality significantly decreased over time, and even there were no major changes in infection-related morbidity. Our data suggest that better education and increasing experience regarding infectious complications were responsible for this improvement. However, future studies have to focus on the reduction of infection-related morbidity, in particular on the reduction of bacterial infections.

## Figures and Tables

**Table 1:** Classification of infectious episodes in pediatric patients with AML treated in the (a) standard-risk (SR) or (b) high-risk (HR) group according to chemotherapy

*(a)*					
*Patients of SR group*	*Total*	*Induction*	*AI*	*haM*	*HAE*
Patients	118	118	110	107	104
Episodes of infection	368	121	66	83	98
FUO	215 (58.4^a^)	72 (60.0)	44 (66.6)	50 (60.3)	49 (50.0)
Clin. documented	43 (11.7)	25 (20.7)	4 (6.1)	3 (3.6)	11 (11.2)
Microb. documented	110 (29.9)	24 (19.8)	18 (27.3)	30 (36.1)	38 (38.8)

*(b)*						
*Patients of HR group*	*Total*	*Induction*	*HAM*	*Consolidation*	*haM*	*HAE*
Patients	287	287	286	261	244	218
Episodes of infection	958	265	189	195	159	150
FUO	529 (55.2^a^)	166 (62.7)	116 (61.3)	95 (48.7)	75 (47.2)	77 (51.3)
Clin. documented	109 (11.4)	34 (12.8)	23 (12.2)	22 (11.3)	17 (10.7)	13 (8.7)
Microb. documented	320 (33.4)	65 (24.5)	50 (26.5)	78 (40.0)	67 (42.1)	60 (40.0)

Abbreviations: AML, acute myeloid leukemia; Clin. documented, clinically documented; FUO, fever of unknown origin; Microb. documented, microbiologically documented.

For specification of chemotherapy see text.

a Percent of infectious episodes.

**Table 2:** Distribution of Gram-negative and Gram-positive bloodstream isolates in pediatric patients with AML treated in the (a) standard-risk (SR) or (b) high-risk (HR) group according to chemotherapy

*(a)*							
*Patients of SR group*	*Total*	*Neutrophils/μl*		*Induction*	*AI*	*haM*	*HAE*
		*<500*	*>500*				
All	87 (100%)	62 (71.3)	25 (28.7)	18 (20.7)	13 (14.9)	28 (32.2)	28 (32.2)
Gram positive	65 (74.7)	48 (55.2)	17 (19.5)	15 (17.2)	13 (14.9)	19 (21.8)	18 (20.7)

*Staphylococci*							
All	16 (18.4)	12 (13.8)	4 (4.6)	6 (6.9)	5 (5.7)	3 (3.4)	2 (2.3)
CoNS	12 (13.8)	9 (10.3)	3 (3.4)	6 (6.9)	3 (3.4)	2 (2.3)	1 (1.1)
S*taphylococcus aureus*	1 (1.1)	0	1 (1.1)	0	0	1 (1.1)	0

*Streptococci*							
All	37 (42.5)	31 (35.6)	6 (6.9)	5 (5.7)	6 (6.9)	13 (14.9)	13 (14.9)
VGS	33 (37.9)	28 (32.2)	5 (5.7)	4 (4.6)	6 (6.9)	13 (14.9)	10 (11.5)
*Enterococcus* spp.	1 (1.1)	0 (0)	1 (1.1)	0	1 (1.1)	0	0
*Micrococcus* spp.	6 (6.9)	2 (2.3)	4 (4.6)	3 (3.4)	1 (1.1)	1 (1.1)	1 (1.1)
Other^a^	5 (5.7)	3 (3.4)	2 (2.3)	1 (1.1)	0	2 (2.3)	2 (2.3)
Gram-negative	22 (25.3)	14 (16.1)	8 (9.2)	3 (3.4)	0	9 (10.3)	10 (11.5)
*Klebsiella* spp.	5 (5.7)	5 (5.7)	0	1 (1.1)	0	1 (1.1)	3 (3.4)
*Pseudomonas aeruginosa*	2 (2.3)	1 (1.1)	1 (1.1)	1 (1.1)	0	1 (1.1)	
*Escherichia coli*	9 (10.3)	8 (9.2)	1 (1.1)	0	0	2 (2.3)	7 (8.0)
*Enterobacter* spp.	0	0	0	0	0	0	0
*Acinetobacter* spp.	0	0	0	0	0	0	0
Other^b^	6 (6.9)	0	6 (6.9)	1 (1.1)	0	5 (5.7)	0

*(b)*								
*Patients of HR group*	*Total*	*Neutrophils/μl*		*Induction*	*HAM*	*Consolidation*	*haM*	*HAE*
		*<500*	*>500*					
All	243 (100%)	176 (72.4)	67 (27.6)	37 (15.2)	29 (11.9)	64 (26.3)	55 (22.6)	58 (23.9)
Gram positive	175 (72.0)	118 (48.6)	57 (23.5)	29 (11.9)	24 (9.9)	43 (17.7)	39 (16.0)	40 (16.5)

*Staphylococci*								
All	54 (22.2)	27 (11.1)	27 (11.1)	11 (4.5)	10 (4.1)	10 (4.1)	12 (4.9)	11 (4.5)
CoNS	41 (16.9)	21 (8.6)	20 (8.2)	7 (2.9)	8 (3.3)	10 (4.1)	10 (4.1)	6 (2.5)
S. *aureus*	5 (2.1)	1 (0.4)	4 (1.6)	1 (0.4)	0	0	1 (0.4)	3 (1.2)

*Streptococci*								
All	93 (38.3)	68 (19.8)	25 (10.3)	12 (4.9)	12 (4.9)	22 (9.1)	23 (9.5)	24 (9.9)
VGS	79 (32.5)	54 (22.2)	25 (10.3)	11 (4.5)	8 (3.3)	20 (8.2)	18 (7.4)	22 (9.1)
*Enterococcus* spp.	5 (2.1)	4 (1.6)	1 (0.4)	2 (0.8)	0	2 (0.8)	0	1 (0.4)
*Micrococcus* spp.	8 (3.3)	6 (2.5)	2 (0.8)	1 (0.4)	1 (0.4)	3 (1.2)	0	3 (1.2)
Other^c^	15 (6.2)	13 (5.3)	2 (0.8)	3 (1.2)	1 (0.4)	6 (2.5)	4 (1.6)	1 (0.4)
Gram-negative	68 (28.0)	58 (23.9)	10 (4.1)	8 (3.3)	5 (2.1)	21 (8.6)	16 (6.6)	18 (7.4)
*Klebsiella* spp.	10 (4.1)	9 (3.7)	1 (0.4)	0	0	5 (2.1)	1 (0.4)	4 (1.6)
*P. aeruginosa*	16 (6.6)	14 (5.8)	2 (0.8)	2 (0.8)	2 (0.8)	4 (1.6)	4 (1.6)	4 (1.6)
*E. coli*	23 (9.5)	20 (8.2)	3 (1.2)	4 (1.6)	0	8 (3.3)	5 (2.1)	6 (2.5)
*Enterobacter* spp.	9 (3.7)	8 (3.3)	1 (0.4)	0	1 (0.4)	1 (0.4)	5 (2.1)	2 (0.8)
*Acinetobacter* spp.	2 (0.8)	2 (0.8)	0 (0.0)	0	1 (0.4)	0	0	1 (0.4)
Other^d^	8 (3.3)	5 (2.1)	3 (1.2)	2 (0.8)	1 (0.4)	3 (1.2)	1 (0.4)	1 (0.4)

Abbreviations: AML, acute myeloid leukemia; CoNS, coagulase-negative staphylococci; VGS, viridans group streptococci.

Polymicrobial bacteremia was seen in 8 patients (7 patients with 2 pathogens, 1 patient with 3 pathogens) in the SR group, and in 11 patients (9 patients with 2 pathogens, 2 patients with 3 pathogens) in the HR group.

For specification of chemotherapy see text.

a Includes *Gemella morbillorum* (*n*=1), Gram-positive cocci (3) and Gram-positive rods (1).

b Includes Gram-negative cocci (1) and Gram-negative rods (5).

c Includes *Gemella morbillorum* (1), Gram-positive cocci (10) and Gram-positive rods (4).

d Includes *Serratia marrescens* (1), *Salmonella* spp. (2), *Stenotrophomonas maltophilia* (1), Gram-negative cocci (1) and Gram-negative rods (4).

**Table 3:** Sites of clinically and microbiologically documented infections in pediatric patients with AML treated in the (a) standard-risk (SR) or (b) high-risk (HR) group according to chemotherapy

*(a)*					
*Patients of SR group*	*Total*	*Induction*	*AI*	*haM*	*HAE*
All	153 (100%)	49 (32.0)	22 (14.5)	33 (21.6)	49 (31.9)
Blood	79 (51.6)	16 (10.5)	11 (7.2)	26 (17.0)	26 (17.0)
Lung^a^	36 (23.5)	17 (11.1)	7 (4.6)	3 (2.0)	9 (5.8)
Gastrointestinal tract^b^	24 (15.7)	9 (5.8)	2 (1.3)	4 (2.6)	9 (5.8)
Soft tissue^c^	13 (8.5)	7 (4.6)	1 (0.7)	0	5 (3.3)
Urinary tract^d^	1 (0.7)	0	1 (0.7)	0	0

*(b)*						
*Patients of HR group*	*Total*	*Induction*	*HAM*	*Consolidation*	*haM*	*HAE*
All	429 (100%)	99 (23.0)	73 (17.0)	100 (23.3)	84 (19.7)	73 (17.0)
Blood	230 (53.6)	34 (7.8)	27 (6.3)	61 (14.2)	53 (12.5)	55 (12.8)
Lung^e^	102 (23.9)	37 (8.7)	26 (6.1)	20 (4.7)	10 (2.3)	9 (2.1)
Gastrointestinal tract^f^	56 (13.0)	22 (5.1)	7 (1.6)	11 (2.6)	12 (2.8)	4 (0.9)
Soft tissue^g^	23 (5.4)	6 (1.4)	8 (1.9)	4 (0.9)	3 (0.7)	2 (0.5)
Urinary tract^h^	13 (3.0)	0	4 (0.9)	3 (0.7)	3 (0.7)	3 (0.7)
Other^i^	5 (1.1)	0	1 (0.2)	1 (0.2)	3 (0.7)	0

Abbreviations: AML, acute myeloid leukemia; CoNS, coagulase-negative staphylococci; VGS, viridans group streptococci.

For specification of chemotherapy see text.

a Includes 4 patients with microbiologically documented pneumonia (probable invasive pulmonary aspergillosis (*n=*3) and viridans group streptococci (1)).

b Gastrointestinal tract infection was caused by *Clostridium difficile* (*n=*11), *Salmonella* spp. (1), rotavirus (5), norovirus (2) and adenovirus (1).

c Includes 4 patients with microbiologically documented soft tissue infection (*Pseudomonas aeruginosa* (1), *Escherichia coli* (1), *Enterococcus faecalis* (1) and *Candida* spp. (1)).

d Urinary tract infection was caused by *Enterococcus* spp. (1).

e Includes 23 patients with microbiologically documented pneumonia (probable/proven invasive pulmonary aspergillosis (*n=*5/2), *Staphylococcus aureus* (1), viridans group streptococci (3), RSV (3), rhinovirus (2), adenovirus (4) and influenza/parainfluenza (3)).

f Gastrointestinal tract infection was caused by *Clostridium difficile* (*n=*24), *Salmonella* spp. (4), rotavirus (7), norovirus (16), adenovirus (3) and astrovirus (2).

g Includes 7 patients with microbiologically documented soft tissue infection ((*P. aeruginosa* (3), *Enterococcus faecalis* (2) and *S. aureus* (3)).

h Urinary tract infection was caused by *Enterococcus* spp. (5), *P. aeruginosa* (1), *E. coli* (3), *Proteus mirabilis* (2) and *S. aureus* (2).

i Includes patients with appendicitis (1) and patients with herpes zoster (4).
